# Effect of various weight loss interventions on serum NT-proBNP concentration in severe obese subjects without clinical manifest heart failure

**DOI:** 10.1038/s41598-021-89426-7

**Published:** 2021-05-12

**Authors:** Tim Hollstein, Kristina Schlicht, Laura Krause, Stefanie Hagen, Nathalie Rohmann, Dominik M. Schulte, Kathrin Türk, Alexia Beckmann, Markus Ahrens, Andre Franke, Stefan Schreiber, Thomas Becker, Jan Beckmann, Matthias Laudes

**Affiliations:** 1 Division of Endocrinology, Diabetes and Clinical Nutrition, Department of Internal Medicine 1, University of Kiel, Arnold Heller Straße 3, 24105 Kiel, Germany; 2 Institute for Clinical Molecular Biology, University of Kiel, Arnold Heller Straße 3, 24105 Kiel, Germany; 3 Department of General and Abdominal Surgery, University of Kiel, Arnold Heller Straße 3, 24105 Kiel, Germany; 4 Helios Klinik Lengerich, Martin-Luther-Straße 49, 49525 Lengerich, Germany

**Keywords:** Obesity, Endocrine system and metabolic diseases

## Abstract

Obesity is associated with a “natriuretic handicap” indicated by reduced N-terminal fragment of proBNP (NT-proBNP) concentration. While gastric bypass surgery improves the natriuretic handicap, it is presently unclear if sleeve gastrectomy exhibits similar effects. We examined NT-proBNP serum concentration in n = 72 obese participants without heart failure before and 6 months after sleeve gastrectomy (n = 28), gastric bypass surgery (n = 19), and 3-month 800 kcal/day very-low calorie diet (n = 25). A significant weight loss was observed in all intervention groups. Within 6 months, NT-proBNP concentration tended to increase by a median of 44.3 pg/mL in the sleeve gastrectomy group (p = 0.07), while it remained unchanged in the other groups (all p ≥ 0.50). To gain insights into potential effectors, we additionally analyzed NT-proBNP serum concentration in n = 387 individuals with different metabolic phenotypes. Here, higher NT-proBNP levels were associated with lower nutritional fat and protein but not with carbohydrate intake. Of interest, NT-proBNP serum concentrations were inversely correlated with fasting glucose concentration in euglycemic individuals but not in individuals with prediabetes or type 2 diabetes. In conclusion, sleeve gastrectomy tended to increase NT-proBNP levels in obese individuals and might improve the obesity-associated “natriuretic handicap”. Thereby, nutritional fat and protein intake and the individual glucose homeostasis might be metabolic determinants of NT-proBNP serum concentration.

## Introduction

B-type natriuretic peptide (BNP) is a hormone from the family of natriuretic peptides involved in salt and water homeostasis and secreted by cardiac myocytes in response to atrial stretch^1^. The production of BNP is a two-step process^2^. First, a 26—amino-acid sequence is cleaved from the preprohormone pre-proBNP between Ser^26^ and His^27^, which generates the 108—amino acid sequence prohormone proBNP. Then, proBNP is cleaved again between Arg^102^ and Ser^103^ into the 32—amino acid sequence active hormone BNP and the 76—amino acid sequence biologically inactive N-terminal fragment of pro BNP (NT-proBNP). Both, BNP and NT-proBNP are released into circulation in equimolar amounts^3^.

B-type natriuretic peptide mediates various physiological effects: it decreases blood pressure by increasing renal electrolyte and water excretion, by peripheral vasodilation, and by regulating vascular endothelial permeability^1^. Additionally, BNP increases lipolysis and energy expenditure in adipose tissue^4^ and suppresses cardiac hypertrophy and fibrosis^5^. B-type natriuretic peptide and NT-proBNP are used as biomarkers in the diagnosis and prognosis of heart failure^3^.

Previous studies have found that BNP and NT-proBNP serum concentrations are inversely associated with obesity^6–15^ and that obese individuals show an attenuated natriuretic peptide response upon saline infusion^16^. Thus, obesity is associated with a “natriuretic handicap”^17^ which might mediate metabolically adverse effects by increasing the risk for developing hypertension^16,18,19^, hypertension-related cardiovascular disorders^6^, and type 2 diabetes^20,21^ The reversal of this “natriuretic handicap” state in obese individuals might reduce their risk to develop or worsen these metabolic comorbidities.

Numerous previous studies^22–28^, but not all^29–31^, reported that weight loss interventions are able to restore adequate BNP and NT-proBNP serum concentrations in obese individuals. Further, there is evidence that weight loss induced by gastric bypass surgery may be a better strategy to increase NT-proBNP (and consequently BNP) compared to a hypocaloric diet^23,25^. Besides gastric bypass surgery, sleeve gastrectomy is another method to induce weight loss^32^. So far, only one recent study reported that BNP increases after sleeve gastrectomy^33^. It is unclear whether sleeve gastrectomy also leads to a similar increase in NT-proBNP compared to gastric bypass surgery or a hypocaloric diet, respectively.

Therefore, in this present study, we investigated the impact of two surgical (sleeve gastrectomy *versus* gastric bypass) and one non-surgical (very low-calorie diet [VLCD]) weight loss interventions on NT-proBNP serum concentration in individuals with obesity and without clinical signs of heart failure. Additionally, in exploratory analyses, we identified determinants of NT-proBNP serum concentration in a cross-sectional study population, using available measures of obesity, blood pressure, macronutrient intake, inflammation markers, and insulin resistance.

## Methods

The intervention study includes data from 72 severely obese individuals (20 men, 52 women, BMI > 37 kg/m^2^) who were treated at the interdisciplinary center of obesity medicine of the University Hospital Schleswig–Holstein (Kiel, Germany), and who were selected to either receive a surgical or non-surgical weight loss therapy based on international guidelines and clinical judgement of the interdisciplinary obesity board. Of the total cohort, n = 28 underwent sleeve gastrectomy, n = 19 gastric bypass surgery, and n = 25 the VLCD. Only patients with complete follow-up data and without clinical heart failure diagnosis were included in this present analysis (Supplemental Fig. 1). Before inclusion into the study, a written confirmed consent was obtained from each subject. The study was conducted according to the guidelines laid down in the Declaration of Helsinki and was approved by the ethic committee of the Medical Faculty of the University of Kiel (Germany).

All patients underwent a fasting blood draw and a body weight and height measurement at baseline, e.g. before surgery and before the start of the VLCD (see study design in Supplemental Fig. 2). Patients in the surgery groups underwent laparoscopic sleeve gastrectomy or Roux-en-Y gastric bypass surgery. For gastric bypass surgery, a pouch length of 5–6 cm with an 18 mm bougie was used, a side-to-side anastomosis was performed using a 45-mm linear stapler, and the achieved biliopancreatic and alimentary limb lengths were 100 cm and 150 cm, respectively, as previously described^34,35^. For sleeve gastrectomy, a 14 mm bougie was used. Out of all 47 individuals who underwent bariatric surgery, n = 5 from the sleeve gastrectomy cohort and n = 3 from the gastric bypass cohort received a structured VLCD program using a liquid diet for 6 to 10 weeks pre-surgery. The decision was based on the surgeon’s clinical judgement. Regardless of receiving a VLCD, all patients were advised to remain weight stable and consume a protein-rich, low-carbohydrate diet.

All patients were examined by a surgeon 1 month post-surgery. After 6 months, blood draws and body weight and height measurements were repeated. The VLCD intervention is presented in detail elsewhere^36^. Briefly, patients in the VLCD group were given an approximately 800 kcal/day formula-based and micronutrient-balanced diet for 3 months with a subsequent transition and weight maintenance phase of further 3 months. After 6 months, blood draws and body weight and height measurements were repeated.

In a second part of this paper, we explored determinants of NT-proBNP concentration in a cross-sectional study population. These exploratory analyses are based on data of a Northern German study population which was recruited as part of the project Food Chain Plus (FoCus, funded by the German Federal Ministry of Education and Research) by the PopGen biobank in Kiel, Germany. At time of analysis, n = 470 individuals aged between 18 and 80 years of the adiposity outpatient clinic of the University Hospital Schleswig–Holstein (UKSH) and from the general population of Kiel using the local population registry or word of mouth were enrolled into the study and analyzed in this present study. Baseline examinations were conducted between 2011 and 2015. Participants were asked to fill-in questionnaires with regard to demographic, lifestyle, and medical history, including the EPIC 12 month food frequency questionnaire^37,38^. Participants with incomplete questionnaire data (n = 22), unavailable NT-proBNP values due to technical issues with the ELISA kit (n = 31), and a preexisting diagnosis of heart failure (n = 30) were excluded (Supplemental Fig. 3). The study was conducted according to the guidelines laid down in the Declaration of Helsinki and was approved by the ethic committee of the Medical Faculty of the University of Kiel (Germany). All participants had given their informed consent prior to participation.

### Anthropometric analyses

In both studies (longitudinal and cross-sectional), body weight was measured with a Tanita Scale (Body Composition Analyzer; Type BC- 418 MA; Tanita Corporation, Tokyo, Japan).

### Laboratory analyses

Blood was drawn in the morning in a fasted state. Serum was stored immediately at − 80 °C. Blood samples underwent routine laboratory analyses at the central laboratory of the University Hospital Schleswig–Holstein (Kiel, Germany), e.g., for measuring C-reactive protein (CRP) and interleukin 6 (IL-6). After determination of fasting insulin by an electro-chemiluminescence immunoassay (ECLIA; Elecsys system; Roche, Basel, Switzerland) and of fasting glucose by a glucose-hexokinase UV test (Hitachi Modular; Roche), the Homeostasis Model Assessment (HOMA) index was calculated as glucose (mg/dl) × insulin (μU/ml) / 405. In the intervention cohort, complete and valid fasting blood glucose data of both pre- and post-time points were available from n = 14 (sleeve gastrectomy), n = 9 (gastric bypass), and n = 25 (VLCD) participants. NT-proBNP was measured using a commercially available ELISA kit (Biomedica Medizinprodukte GmbH, Wien, KAT.NR. SK-1204) with an intra- and inter-assay CV of ≤ 4% and ≤ 7%. In the intervention group, 58% of NT-proBNP values were below the assay’s detection limit of 25.4 pg/mL (n = 2 values were 0 pg/mL, n = 38 were 8.5 pg/mL [which corresponds to 1 pmol/L], and n = 2 values were between 8.5 and 25.4 pg/mL). In the cross-sectional group, 57% of NT-proBNP values were below detection limit (n = 35 values were 0 pg/mL, n = 167 were 8.5 pg/mL, and n = 18 values were between 8.5 and 25.4 pg/mL). Tryptophan serum levels were measured by liquid chromatography and tandem mass spectrometry (Agilent 1100 HPLC/CTC-PAL Autosampler/Sciex API 4000 Triple Quadrupole) in an external specialized laboratory (Medizinisches Labor Bremen).

### Statistical analysis

Statistical data analysis was performed using the SAS statistical software package (SAS Enterprise Guide Version 7.15; SAS Institute, Cary, NC).

Power calculations were performed prior to analyses to calculate the minimum detectable significant increase in NT-proBNP after bariatric surgery, based on a previous study with similar design^25^. Assuming a power of 0.80 and a 2-sided alpha level of 0.05, the sample size required to detect a significant increase in NT-proBNP after bariatric surgery was n = 19, which is comparable to the sample sizes of the intervention groups of this paper.

Unless otherwise specified, continuous parameters are presented as mean ± SD and were compared using paired or unpaired student t tests. Continuous parameters not normally distributed are presented as median (25th – 75th percentiles) and were compared using Wilcoxon signed rank tests. For multiple comparisons of quantitative variables with normal and non-normal distributions we used one-way ANOVA and Kruskal–Wallis tests, respectively. The χ^2^ test was used to compare categorical data. All individual changes (∆) in weight and NT-proBNP serum levels were calculated as the difference between post- minus pre-treatment and analyzed by Student’s paired t-tests when data was normally distributed and by Wilcoxon signed rank test when data was not normally distributed. Changes between post- minus pre-treatment are presented as mean with 95% confidence interval (CI) when data was normally distributed. The Pearson’s correlation coefficient was used to quantify associations between continuous variables.

Due to the skewed distribution of NT-proBNP values in the interventional cohort (n = 42 below the ELISA detection limit of 25.4 pg/mL), only NT-proBNP values above the detection limit (n = 30) were used in the main analysis. Sensitivity analyses were performed using all available NT-proBNP values including those below the ELISA detection limit (n = 72).

Due to the skewed distribution of NT-proBNP values in the cross-sectional FoCus cohort (n = 220 values below the ELISA detection limit), we created three groups representing 1) NT-proBNP concentration below detection limit (n = 220) as well as 2) *low* and 3) *high* NT-proBNP concentration. The latter two groups were created based on the median (158.9 pg/mL) of all detectable values. Individuals with an NT-proBNP concentration above 25.4 pg/mL but below 158.9 pg/mL were assigned to the *low* NT-proBNP group (n = 83), while individuals with an NT-proBNP concentration equal or above 158.9 pg/mL were assigned to the *high* NT-proBNP group (n = 84). To determine associations between NT-proBNP groups and other variables, p trend analyses were performed using a linear regression model with NT-proBNP group as ordinal covariate and the variable of interest as dependent variable. In addition, sensitivity analyses were performed in the whole cohort (n = 387) by using non-parametric Spearman correlation analyses which do not rest upon an assumption of normality.

## Results

### Change in NT-proBNP serum concentration after three different weight loss interventions

Baseline characteristics of the intervention groups are presented in Table 1. Briefly, all three intervention groups mainly consisted of females (76.0–78.9%) and mean age ranged from 43.3–50.6 years. The intervention groups were not statistically different in terms of baseline body weight (mean range 134.3–155.5 kg, p = 0.07), BMI (mean range 46.2–50.8 kg/m^2^, p = 0.07), fasting blood glucose (mean range 111.6–147.2 mg/dL, p = 0.12), and NT-proBNP concentration (median range 94.4–189.7 pg/mL, p = 0.72).

**Table 1:** Baseline characteristics of intervention groups.

	Sleeve gastrectomy n = 28	Gastric bypass n = 19	VLCD n = 25
Male (%)	9 (32.1)	5 (21.1)	6 (24.0)
Diabetes mellitus (%)	10 (35.7)	5 (26.3)	6 (24.0)
Age (years)	43.9 ± 9.6 (22.0, 65.0)	43.3 ± 9.3 (27.0, 63.0)	50.6 ± 11.6 (30.0, 69.0)
Height (cm)	1.74 ± 0.11 (1.57, 2.05)	1.74 ± 0.13 (1.60, 2.08)	1.70 ± 0.08 (1.54, 1.90)
Weight (kg)	155.5 ± 43.1 (107.0, 275.0)	145.5 ± 29.4 (99.0, 222.0)	134.3 ± 18.6 (99.4, 165.3)
BMI (kg/m^2^)	50.8 ± 9.2 (40.2, 74.9)	48.1 ± 6.7 (37.3, 58.6)	46.2 ± 4.2 (40.2, 55.8)
Fasting blood glucose (mg/dL)^1^	133.1 ± 59.9 (97.0, 328.0)	147.2 ± 69.0 (95.0, 312.0)	111.6 ± 26.7 (77.0, 195.0)
NT-proBNP (pg/mL)^2,3^	170.7; 75.4–284.5 (36.0, 764.4)	189.7; 63.4–272.5 (41.1, 317.5)	94.4; 72.3–179.3 (46.2, 570.8)

Values are presented as mean ± SD for continuous variables or number (frequency) for categorical variables with minimum and maximum in parentheses. ^1^Only complete and valid fasting blood glucose values were included in the analysis (sleeve gastrectomy: n = 14, gastric bypass n = 9, VLCD: n = 25). ^2^Skewed values are expressed as medians with interquartile ranges separated by a hyphen and with minimum and maximum in parentheses. ^3^Only NT-proBNP values above the ELISA detection limit were included in the analysis (sleeve gastrectomy: n = 14, gastric bypass n = 7, VLCD: n = 9).

BMI, body mass index; NT-proBNP, N-terminal pro brain natriuretic peptide; VLCD, very low-calorie diet.

**Figure 1:**
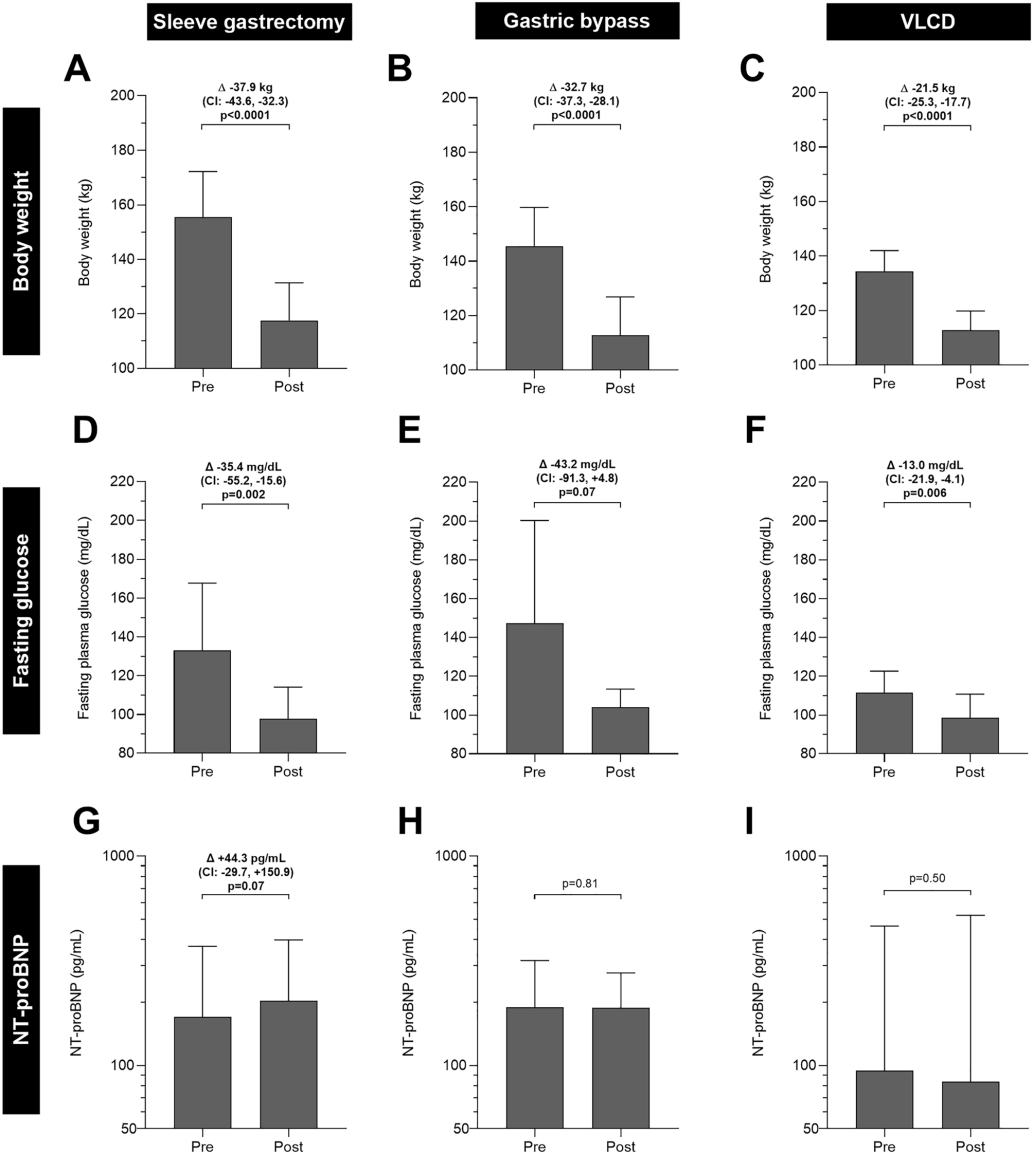
Comparison of weight loss and concomitant changes in fasting blood glucose and NT-proBNP concentration after sleeve gastrectomy, gastric bypass surgery, and a 26-week weight loss program. Left column denotes pre- and post (**A**) weight, (**D**) fasting blood glucose, and (**G**) NT-proBNP concentration after 26 weeks of sleeve gastrectomy follow-up. Middle column denotes pre- and post (**B**) weight, (**E**) fasting blood glucose, and (H) NT-proBNP concentration after 26 weeks of gastric bypass surgery follow-up. Right column denotes pre- and post (**C**) weight, (**F**) fasting blood glucose, and (**I**) NT-proBNP concentration after 13 weeks of VLCD + 13 weeks of weight maintenance. All three intervention groups were not statistically different in terms of baseline NT-proBNP concentration (p = 0.72). In panels (**A**–**F**), bars denote mean weight and error bars denote 95% CI of the mean. Statistical significance of changes in weight and fasting blood glucose from pre- to post-intervention was determined by Student’s paired t-test. Only complete and valid fasting blood glucose values were included in the analysis (sleeve gastrectomy: n = 14, gastric bypass n = 9, VLCD: n = 25). In panels G–I, bars denote median NT-proBNP concentration and error bars denote 95% CI of the median. Y axes are formatted in log_10_ to account for skewed distribution of NT-proBNP values. Only NT-proBNP values above the ELISA detection limit were included in the analysis (sleeve gastrectomy: n = 14, gastric bypass n = 7, VLCD: n = 9). Statistical significance of changes in NT-proBNP concentration from pre- to post-intervention was determined by Wilcoxon signed rank test. CI; confidence interval; NT-proBNP, N-terminal pro brain natriuretic peptide; VLCD, very low-calorie diet.

After 26-weeks, participants who underwent sleeve gastrectomy lost on average 37.9 kg (95% CI: –43.6, –32.3, p < 0.0001, Fig. 1A), participants who underwent gastric bypass surgery lost on average 32.7 kg (95% CI: –37.3, –28.1, p < 0.0001, Fig. 1B), and VLCD participants lost on average 21.5 kg (95% CI: –25.3, –17.7, p < 0.0001, Fig. 1C). Weight loss was greater in both bariatric surgery groups compared to VLCD (both p < 0.007) while there was no difference between sleeve gastrectomy and gastric bypass surgery (p = 0.29).

Weight loss was accompanied by reductions in fasting blood glucose by –35.4 mg/dL (95% CI: –55.2, –15.6, p = 0.002, Fig. 1D) in the sleeve gastrectomy group, by –43.2 mg/dL (95% CI: –91.3, + 4.8, p = 0.07, Fig. 1E) in the gastric bypass group, and by –13 mg/dL (95% CI: –21.9, –4.1, p = 0.006, Fig. 1F) in the VLCD group. Reductions in fasting blood glucose were comparable between both bariatric interventions (p = 0.70), while sleeve gastrectomy and gastric bypass surgery led to greater fasting blood glucose reduction than VLCD (both p ≤ 0.04). Weight loss after VLCD was associated with concomitant reductions in fasting blood glucose (r = 0.48, p = 0.01, Supplemental Fig. 4), while no such associations were found for both bariatric procedures (both p > 0.13).

In the same 26-week period, NT-proBNP concentration increased by a median of 44.3 pg/mL from 170.7 pg/mL (75.4–284.5) to 203.9 pg/mL (136.0–378.8) in the group receiving sleeve gastrectomy (p = 0.07, Fig. 1G), while it remained unchanged in the gastric bypass surgery and VLCD groups (both p ≥ 0.50, Fig. 1H,I). Similar results were obtained in sensitivity analyses when including individuals with a baseline NT-proBNP concentration below the ELISA detection limit of 24.5 pg/mL (Supplemental Fig. 5a-c).

In univariate correlation analyses, there were no associations between changes in weight, BMI, or fasting blood glucose and concomitant changes in NT-proBNP concentration after all three weight loss interventions (all p > 0.50).

### Exploratory analyses of NT-proBNP determinants in a cross-sectional study population

In exploratory analyses, we investigated determinants of NT-proBNP serum concentration in a larger population using data from the cross-sectional FoCus cohort to gain potential mechanistic insights. Characteristics of the FoCus cohort are shown in Table 2. Briefly, the cohort consisted of 387 individuals (72.9% females) without clinical diagnosis of heart failure. Mean age was 53.4 ± 11.7 years, mean BMI was 32.1 ± 12.0 kg/m^2^, and mean systolic/diastolic blood pressure were 129.0 ± 13.7 and 80.7 ± 7.5 mmHg, respectively. The cohort comprised non-diabetic individuals with normal fasting glucose (euglycemic, n = 218) as well as prediabetic (n = 108) and diabetic (n = 61) individuals. Accordingly, mean fasting glucose was 108.5 ± 39.6 mg/dL and ranged from 67.0 to 387.0 mg/dL. Median NT-proBNP was 8.5 (8.5–131.0) pg/mL and significantly higher in females compared to males (17.4 [8.5–153.1] pg/mL vs. 8.5 [8.5–50.7] pg/mL, p = 0.0003).

**Table 2:** Characteristics of the FoCus cohort.

	Total n = 387	Male n = 105	Female n = 282
Age (years)	53.4 ± 11.7 (21.0, 77.0)	56.3 ± 10.4 (30.0, 76.0) *	52.4 ± 12.1 (21.0, 77.0) *
Diabetes mellitus (%)	61 (15.8)	24 (22.9)	37 (13.1) *
**Anthropometrics**			
Height (cm)	171.7 ± 9.0 (150.0, 207.0)	181.3 ± 7.3 (167.0, 207.0) *	168.1 ± 6.7 (150.0, 188.0) *
Weight (kg)	94.8 ± 37.0 (43.4, 203.8)	108.8 ± 38.5 (51.0, 198.7) *	89.6 ± 35.1 (43.4, 203.8) *
BMI (kg/m^2^)	32.1 ± 12.0 (14.3, 70.5)	33.0 ± 11.0 (16.1, 63.9)	31.7 ± 12.3 (14.3, 70.5)
Waist circumference (cm)	105.6 ± 28.6 (59.0, 150.0)	105.3 ± 28.7 (59.0, 150.0)	105.8 ± 28.7 (60.0, 150.0)
**Blood pressure measures**			
Systolic blood pressure (mmHg)	129.0 ± 13.7 (95.0, 220.0)	128.9 ± 13.1 (100.0, 155.0)	129.0 ± 13.9 (95.0, 220.0)
Diastolic blood pressure (mmHg)	80.7 ± 7.5 (60.0, 110.0)	81.2 ± 7.2 (70.0, 95.0)	80.5 ± 7.6 (60.0, 110.0)
**Measures of insulin resistance**			
Fasting glucose (mg/dL)	108.5 ± 39.6 (67.0, 387.0)	116.0 ± 39.1 (68.0, 329.0) *	105.6 ± 39.5 (67.0, 387.0) *
Fasting insulin (µIU/mL)^1^	10.4; 6.1–21.1 (0.2, 403.8)	12.5; 7.1–26.0 (0.4, 403.8)	9.9; 5.8–19.5 (0.2, 243.8)
HOMA-IR (ratio)^1^	2.5; 1.4–5.8 (0.1, 147.6)	3.2; 1.7–6.8 (0.3, 147.6) *	2.3; 1.3–5.2 (0.1, 99.3) *
**Inflammation markers**			
IL-6 (pg/mL)^1^	3.6; 1.9–5.5 (1.5, 75.2)	4.0; 2.1–5.7 (1.5, 75.2)	3.5; 1.9–5.3 (1.5, 30.8)
CRP (mg/L)^1^	2.1; 0.9–6.7 (0.9, 64.5)	1.9; 0.9–5.0 (0.9, 43.7)	2.2; 0.9–6.8 (0.9, 64.5)
Tryptophan (mg/dL)	1.63 ± 0.31 (0.80, 2.69)	1.73 ± 0.32 (0.80, 2.68) *	1.60 ± 0.30 (0.85, 2.69) *
**Natriuretic peptides**			
NT-proBNP (pg/mL)^1^	8.5; 8.5–131.0 (0.0, 6,439.8)	8.5; 8.5–50.7 (0.0, 4,803.6) *	17.4; 8.5–153.1 (0.0, 6,439.8) *

Values are presented as mean ± SD for continuous variables or number (frequency) for categorical variables with minimum and maximum in parentheses. ^1^Skewed values are expressed as medians with interquartile ranges and with minimum and maximum in parentheses. Statistical significance between males and females was determined with unpaired t test for normally distributed data, with Wilcoxon test for skewed data, and with χ^2^ test for categorical data. An asterisk (*) denotes significant differences between both groups (p < 0.05).

BMI, body mass index; CRP, C-reactive protein; HOMA-IR, homeostatic model assessment of insulin resistance; IL-6, interleukin 6; NT-proBNP, N-terminal pro brain natriuretic peptide.

**Figure 2:**
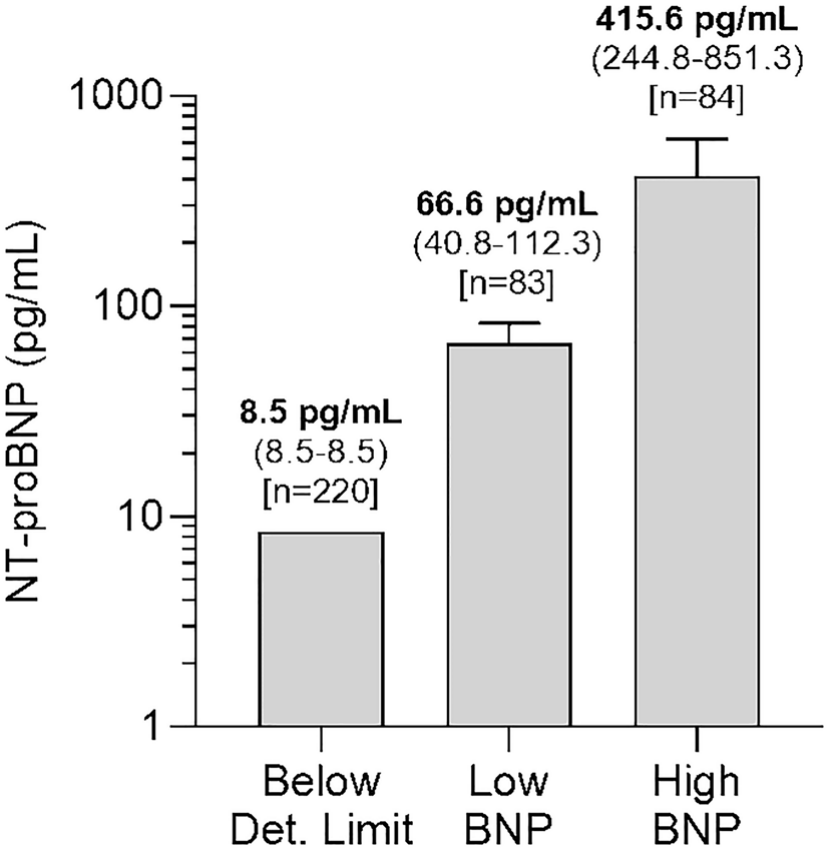
Definition of NT-proBNP groups based on NT-proBNP plasma concentration in cross-sectional analysis. Due to the skewed distribution of NT-proBNP values in the cross-sectional FoCus cohort, we created three groups representing (1) NT-proBNP concentration below detection limit of 25.4 pg/mL (n = 220, left bar) as well as (2) *low* and (3) *high* NT-proBNP concentration. The latter two groups were created based on the median (158.9 pg/mL) of all detectable values. Individuals with an NT-proBNP concentration above 25.4 pg/mL but below 158.9 pg/mL were assigned to the *low* NT-proBNP group (n = 83, middle bar), while individuals with an NT-proBNP concentration equal or above 158.9 pg/mL were assigned to the *high* NT-proBNP group (n = 84, right bar). Bars denote median NT-proBNP concentration and error bars denote 95% CI of the median. Y axis is formatted in log_10_ to account for skewed distribution of NT-proBNP values. CI; confidence interval; NT-proBNP, N-terminal pro brain natriuretic peptide.

Due to the skewed distribution of NT-proBNP values, participants were grouped into three categories based on their measured NT-proBNP value, representing 1) NT-proBNP concentration below assay detection limit of 25.4 pg/mL (n = 220), 2) *low* NT-proBNP concentration (n = 83 with median NT-proBNP of 66.6 [40.8–112.3] pg/mL), and 3) *high* NT-proBNP concentration (n = 84 with median NT-proBNP of 415.6 [244.8–851.3] pg/mL, Fig. 2).

**Figure 3:**
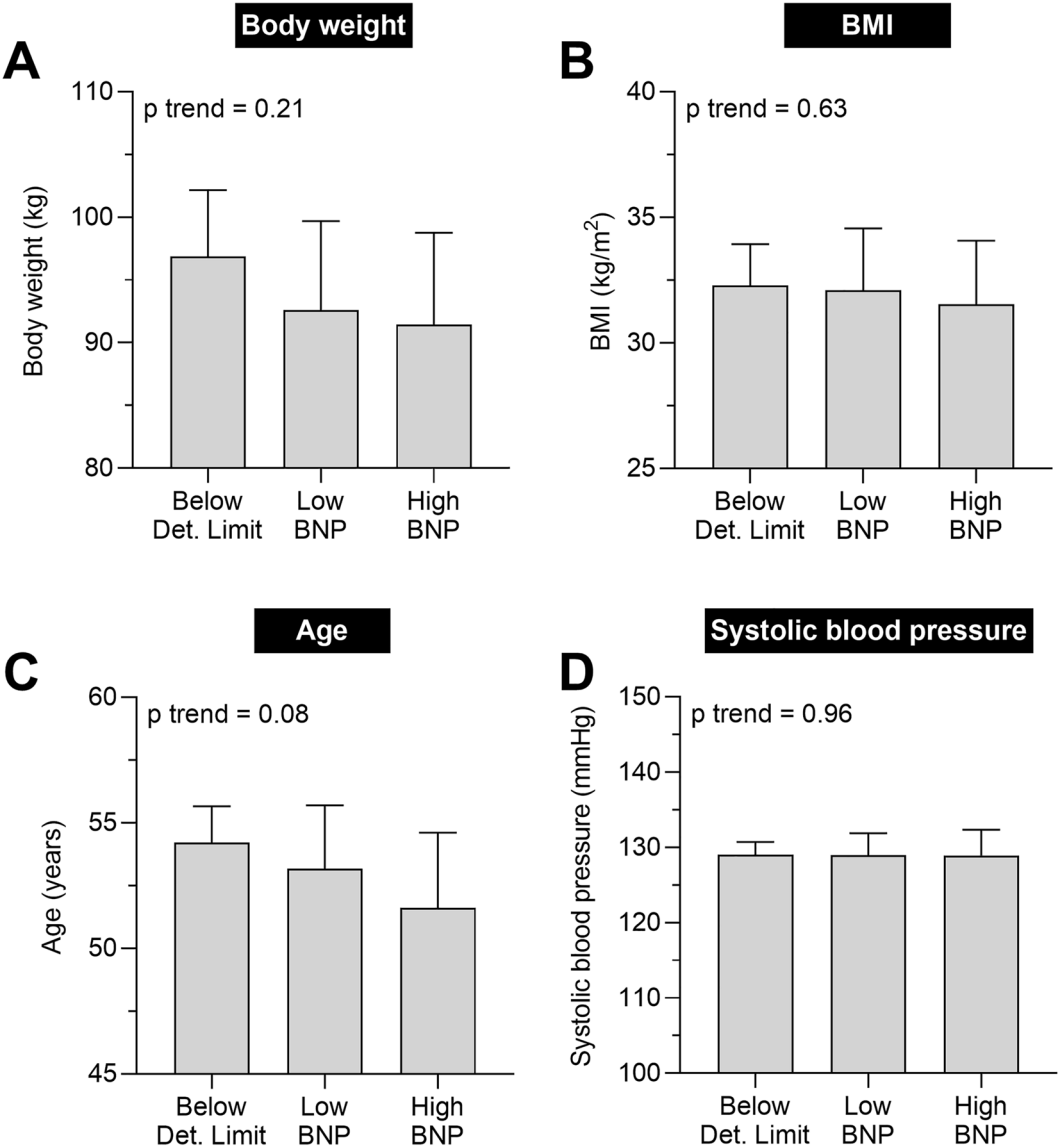
Associations between NT-proBNP concentration and (**A**) body weight, (**B**) BMI, (**C**) age, and (**D**) systolic blood pressure. Bars denote mean and error bars denote 95% CI of the mean. P trend analyses were performed using a linear regression model with NT-proBNP group as ordinal covariate and the variable of interest as dependent variable. Definition of NT-proBNP groups is shown in Fig. 2. BMI, body mass index; CI; confidence interval; NT-proBNP, N-terminal pro brain natriuretic peptide.

Using these groups in statistical analyses, we found no association between NT-proBNP concentration and body weight (p trend = 0.21, Fig. 3A), BMI (p trend = 0.63, Fig. 3B), age (p trend = 0.08, Fig. 3C), and systolic blood pressure (p trend = 0.96, Fig. 3D).

**Figure 4:**
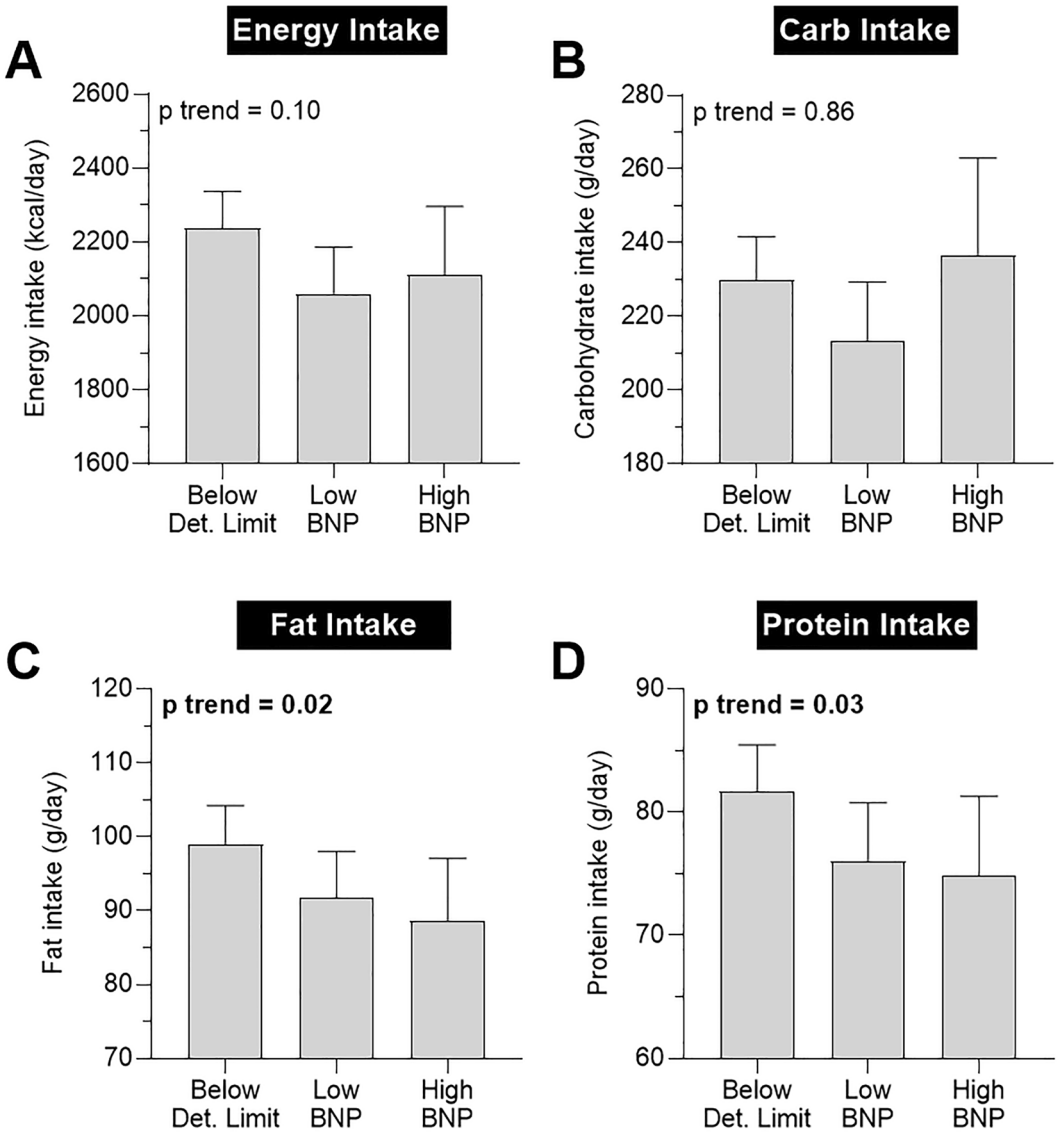
Associations between NT-proBNP concentration and total energy and macronutrient intake. Association between NT-proBNP concentration and (**A**) energy intake, (**B**) carbohydrate intake, (**C**) fat intake, and (**D**) protein intake. Information about the nutritional intake was assessed by a self-reported 12-month nutritional, retrospective (food frequency) questionnaire. Bars denote mean and error bars denote 95% CI of the mean. P trend analyses were performed using a linear regression model with NT-proBNP group as ordinal covariate and the variable of interest as dependent variable. Definition of NT-proBNP groups is shown in Fig. 2. CI; confidence interval; NT-proBNP, N-terminal pro brain natriuretic peptide.

However, high NT-proBNP concentration was associated with less fat intake (p trend = 0.02, Fig. 4C) and less protein intake (p trend = 0.03, Fig. 4D). These results remained significant after further adjustment for BMI (both p ≤ 0.05). There was no trend for decreased carbohydrate and total energy intake with increasing NT-proBNP concentration (both p trend ≥ 0.10, Figs. 4A,B).

**Figure 5:**
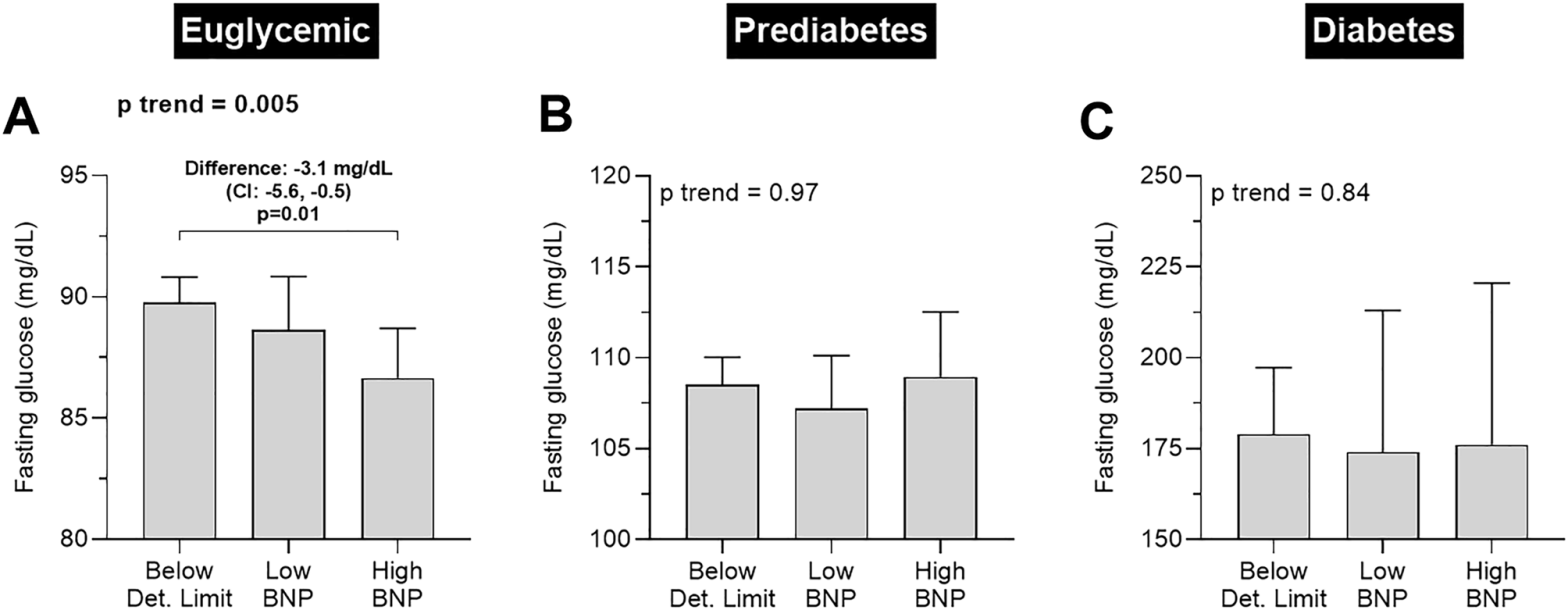
Subgroup analysis: associations between NT-proBNP concentration and fasting plasma glucose in (**A**) euglycemic, (**B**) prediabetic, and (**C**) diabetic individuals. Euglycemia was defined as fasting plasma glucose < 100 mg/dL, prediabetes was defined as fasting plasma glucose between 100 and 126 mg/dL, and diabetes was defined as fasting plasma glucose > 126 mg/dL. Bars denote mean and error bars denote 95% CI of the mean. P trend analyses were performed using a linear regression model with NT-proBNP group as ordinal covariate and the variable of interest as dependent variable. Statistical significance of between-group differences was calculated by unpaired t test. Definition of NT-proBNP groups is shown in Fig. 2. CI; confidence interval; NT-proBNP, N-terminal pro brain natriuretic peptide.

There was no association between NT-proBNP serum concentration and fasting glucose serum concentration (p trend = 0.14). However, when grouping participants based on normal (< 100 mg/dL), prediabetic (between 100 and 126 mg/dL) and diabetic (> 126 mg/dL) fasting glucose, in euglycemic individuals, a higher NT-proBNP concentration was associated with lower fasting blood glucose (p trend = 0.005). In a between-group analysis, euglycemic individuals with high NT-proBNP values had a 3.1 mg/dL (CI: –5.6, –0.5, p = 0.01) lower fasting blood glucose compared to individuals with no detectable NT-proBNP (Fig. 5A). This association remained significant after further adjustment for BMI (p trend = 0.01). The association was not found in prediabetic or diabetic individuals (both p trend > 0.8, Fig. 5B,C). There were no associations between NT-proBNP serum concentration and fasting insulin or HOMA-IR in the whole cohort (all p trend > 0.14) and in euglycemic, prediabetic, and diabetic subgroups (all p trend > 0.07). There was also no association between NT-proBNP concentration and measures of inflammation such as CRP, IL-6, and tryptophan (all p trend ≥ 0.30).

For all presented results, sensitivity analyses were performed by using Spearman correlation analyses in the whole cohort and similar results were obtained (data not shown) except for tryptophan, were an inverse association has been found (r = –0.11, p = 0.03).

## Discussion

In this present study including 72 severely obese individuals with relatively low NT-proBNP serum concentration, we found that sleeve gastrectomy tended to increase NT-proBNP after 6 months of follow-up, while NT-proBNP concentration remained unchanged after gastric bypass surgery and VLCD. Blood glucose was reduced during all 3 interventions. In exploratory, cross-sectional analyses in a cohort of n = 387 individuals, we found that NT-proBNP was higher in females and that higher NT-proBNP concentration was associated with less fat intake as well as with lower fasting glucose in individuals with normal glucose regulation. There was no association between NT-proBNP and measures of obesity and inflammation markers.

### Sleeve gastrectomy might improve the “natriuretic handicap” of severely obese individuals

Natriuretic peptides, including BNP (and its N-terminal counterpart NT-proBNP) can be considered as antagonists of the renin–angiotensin–aldosterone system (RAAS) as they decrease blood pressure, fluid volume and exert antifibrotic and antihypertrophic effect in the heart^1^.

Previous studies reported that obesity is associated with decreased BNP and NT-proBNP concentrations^6–14^, possibly due to attenuated natriuretic peptide secretion^16^ and/or increased clearance as a result of obesity-associated glomerular hyperfiltration^39^. Therefore, individuals with obesity seem to have a “natriuretic handicap”^17^ which exposes them to greater risk for developing hypertension^16,18,19^, hypertension-related cardiovascular disorders^6^, and type 2 diabetes^20,21^.

Weight loss interventions can reverse this “natriuretic handicap”^22–27^, although some studies also reported decreased BNP and NT-proBNP concentrations after weight loss^29–31^. However, these latter studies may be confounded by concomitant reductions in plasma volume and blood pressure, themselves being strong determinants of natriuretic peptide levels^40^.

Interestingly, in one study, weight loss induced by Roux-en-Y gastric bypass surgery increased BNP and NT-proBNP concentrations to a greater extent when compared to weight loss induced by a VLCD^23^, suggesting a weight-loss independent mechanism that improves natriuretic peptide release, possibly by yet unidentified alterations of the neuronal/hormonal balance due to the “disconnected” stomach and duodenum^25^. In contrast, another bariatric surgery technique, gastric banding, has been shown to reduce NT-proBNP in obese individuals^31^.

To date, banding surgery techniques are less and less used in favor of sleeve gastrectomy which reduces the stomach to about 15% of its original size to achieve weight loss and achieves better outcomes and has less complications^32^. So far, no study investigated the change in NT-proBNP after sleeve gastrectomy.

Therefore, in this present study, we compared sleeve gastrectomy to gastric bypass surgery and VLCD and found that sleeve gastrectomy tended to increase NT-proBNP by a median of 44.3 pg/mL (p = 0.07) in severely obese individuals after 6 months of follow-up and an average weight loss of 38 kg. These data support the idea that the “natriuretic handicap” of obese individuals can be improved with surgically-induced weight loss, especially sleeve gastrectomy. The median increase of 44.3 pg/mL is comparable to previous data from gastric bypass surgery showing an increased NT-proBNP concentration by ~ 24–46 pg/ml 6 months after surgery^23,25,27^.

Gastric bypass surgery and VLCD did not significantly increase NT-proBNP concentration (all p ≥ 0.50). This is different from previous studies showing increased NT-proBNP after gastric bypass surgery^23,25,27^ and a hypocaloric diet^22–24^, although NT-proBNP did not change following gastric bypass surgery in another study^41^. As possible confounding variables like sex, age, and BMI were not significantly different between the interventional groups, we hypothesize that other, not measured, determinants (left ventricular hypertrophy, systolic blood pressure, pulse rate) might have contributed to the divergent results between the interventional groups. Furthermore, our finding might have been influenced by a selection bias, that is, patients with higher BMI were more likely to receive sleeve gastrectomy while patients with lower BMI were more likely to receive gastric bypass or VLCD treatment. Lastly, weight loss tended to be greater in the sleeve gastrectomy cohort compared to the gastric bypass cohort, which is different from some^42,43^ but not all^44,45^ studies of similar duration and might have contributed to the greater increase in NT-proBNP in the sleeve gastrectomy cohort.

While we cannot provide a physiological explanation why NT-proBNP increased with sleeve gastrectomy only, we hypothesize that concomitant changes in vasoactive hormones might have contributed to the increase in NT-proBNP after sleeve gastrectomy. Accordingly, one recent study reported that aldosterone, angiotensinogen, angiotensin II, bradykinin, endothelin, neprilysin, and renin were significantly reduced after sleeve gastrectomy while natriuretic peptides significantly increased^33^.

Our results merit further verification in future studies primarily designed to compare different surgical weight loss strategies with regard to changes in natriuretic peptides, including natriuretic peptide type A (ANP).

Interestingly, we also found that fasting blood glucose was reduced during all 3 weight loss interventions which confirms previous studies^25,28,46^. We also report that the VLCD-induced reduction in fasting blood glucose was associated with the concomitant amount of weight loss, which is comparable to a previous study^47^.

### Exploratory analyses: determinants of NT-proBNP concentration

In order to explore potential nutritional and metabolic factors contributing to the above-mentioned effects in this present study, we also investigated which demographic, anthropometric, and metabolic characteristics are associated with NT-proBNP concentration in a cross-sectional cohort of 387 individuals. While we found that NT-proBNP is higher in women compared to men—which is in line with previous studies^15,23,48^ and mediated by lower circulating androgens^48^—we did not find an association between NT-proBNP and measures of obesity. This is different from previous studies which consistently reported this association^6–11,15^ and might be due to, again, the relatively lower NT-proBNP concentration in our cohort (median 8,5 pg/mL) and the fact that our cohort did, by design, not include individuals with a BMI from 25 to 30 kg/m^2^ which might have further influenced this association. Similarly, we did not find an association between NT-proBNP and inflammation markers. In previous studies, there is conflicting evidence whether natriuretic peptides are higher or lower in a proinflammatory state^17,49^.

Interestingly, lower NT-proBNP concentration was associated with higher fat intake in our cohort. Studies investigating the associations between natriuretic peptides and macronutrient intake are scarce, however, one study reported that a traditional Mediterranean high-fat diet decreased NT-proBNP to a greater extent compared to a low-fat control diet^50^. Similarly, in another study, a high-fat diet resulted in lower BNP concentration due to increased circulating levels of neprilysin^51^, an enzyme implicated in BNP degradation^51^. These data—including ours—indicate that low-fat diets may be suitable to reverse the metabolic handicap in individuals with obesity and might explain the effects of weight loss interventions. Future longitudinal studies are warranted to investigate this hypothesis. We also found an inverse association between protein intake and NT-proBNP level in our cohort, e.g. lower protein intake was associated with a higher NT-proBNP concentration. We could not find studies investigating the association between protein intake and NT-proBNP. But higher NT-proBNP has been associated with sarcopenia in patients with type 2 diabetes which supports our data^52^. Carbohydrate intake had no effect on NT-proBNP level in our cohort, which has been reported previously^53^.

Interestingly, in euglycemic individuals, we found that higher NT-proBNP concentration was associated with lower fasting glucose, such that individuals with *high* NT-proBNP concentration (median 415.6 pg/mL) had a lower fasting glucose by 3.1 mg/dL compared to individuals with an NT-proBNP concentration below assay detection limit of 25.4 pg/mL. Our findings are supported by data from the general population^15^ and by a study showing that BNP infusion lowers plasma glucose after a glucose load in healthy, euglycemic men^54^. In that latter study, the authors hypothesized that the BNP-mediated decrease in glucose is due to increased glucose distribution but not due to increased cellular glucose uptake^54^. In contrast to euglycemic individuals, higher BNP is not related to better glucose tolerance in individuals with diabetes^55^, which supports our data. We did not find an association between NT-proBNP and measures of insulin resistance, however, previous studies reported that NT-proBNP was inversely related to insulin resistance^13^ and hyperinsulinemia^15^.

### Limitations

In our study we show for the first time that (i) sleeve gastrectomy is able to increase NT-pro-BNP concentration and (ii) that NT-pro-BNP concentration is related to nutritional fat and protein intake as well as glucose homeostasis in euglycemic individuals. However, some limitations have to be considered: We did not perform echocardiography in both the intervention and cross-sectional cohorts, and therefore, could not adjust NT-proBNP values for echocardiographic variables such as left ventricular mass and left atrial size. Additionally, we did not measure BNP in our cohort, which has been reported to increase after surgically induced weight loss^33^. Further, NT-proBNP concentration was relatively low in both of our cohorts with around 58% of values below the assay’s detection limit of 25.4 pg/mL, which may be due to concomitant drug treatment (which was not assessed in this cohort) and which have influenced the results. However, sensitivity analyses were performed considering only values above the detection limit and similar results were found. In the interventional cohort, we did not find an association between changes in NT-proBNP and weight, BMI, or fasting glucose while some^22,23,28^ but not all^24,25^ interventional weight loss studies reported these associations.

## Conclusion

Surgically induced weight loss by sleeve gastrectomy tended to increase NT-proBNP in individuals with severe obesity and thus might improve their “natriuretic handicap”. In a cross-sectional analysis, higher NT-proBNP was associated with less fat and protein intake and with lower fasting glucose in euglycemic individuals, suggesting that nutritional fat and protein intake as well as the individual glucose homeostasis might be metabolic determinants in regulating serum NT-pro-BNP concentration.

## Supplementary Information

Supplementary Information.

## Abbreviations

BMI: Body mass index
BNP: B-type natriuretic peptide
CRP: C-reactive protein
HOMA: Homeostasis model assessment
IL-6: Interleukin 6
NT-proBNP: N-terminal pro brain natriuretic peptide
VLCD: Very low-calorie diet
