# Supplementary material for: Effect of various weight loss interventions on serum NT-proBNP concentration in severe obese subjects without clinical manifest heart failure
Source: Sci Rep. 2021 May 12;11:10096. doi: 10.1038/s41598-021-89426-7 (PMC8115663; doi:10.1038/s41598-021-89426-7)
Supplement: Supplementary file 1 — Supplementary Information. [file 41598_2021_89426_MOESM1_ESM.docx]

**Supplemental Material**

**Hollstein et al. “Effect of various weight loss interventions on serum NT-proBNP concentration in severe obese subjects without clinical manifest heart failure”**

Tim Hollstein^1^; Kristina Schlicht^1^; Laura Krause^1^; Stefanie Hagen^1^; Nathalie Rohmann^1^; Dominik M. Schulte^1^, Kathrin Türk^1^, Alexia Beckmann^1^, Markus Ahrens^5^, Andre Franke^2^, Stefan Schreiber^1,2^, Thomas Becker^3^, Jan Beckmann^3^, Matthias Laudes^1^

^1^Division of Endocrinology, Diabetes and Clinical Nutrition, Department of Internal Medicine 1, ^2^Institute for Clinical Molecular Biology, ^3^Department of General and Abdominal Surgery,

University of Kiel, Arnold Heller Straße 3, Kiel 24105, Germany.

^5^Helios Klinik Lengerich, Martin-Luther-Straße 49, 49525 Lengerich, Germany

**Corresponding author:** Prof. Dr. Matthias Laudes, Division of Endocrinology, Diabetes and Clinical Nutrition, Department of Internal Medicine 1, University of Kiel, Arnold Heller Straße 3, Kiel 24105, Germany. Email: [matthias.laudes@uksh.de](mailto:matthias.laudes@uksh.de)

**Running title:** NT-proBNP tends to increase after sleeve gastrectomy

**Figures & Tables:** 2 Tables, 5 Figures

**Supplemental Material:** 5 Figures

**Word count:** 4177

**Acknowledgements:** No external funding was used in the conduct of this study.

**Funding:** Faculty means

**Abbreviations:** BMI, body mass index; BNP, B-type natriuretic peptide; CRP, C-reactive protein; HOMA, Homeostasis Model Assessment; IL-6, interleukin 6; NT-proBNP, N-terminal pro brain natriuretic peptide; VLCD, very low-calorie diet.

## Supplemental Figure 1 – Study flowchart of the intervention groups


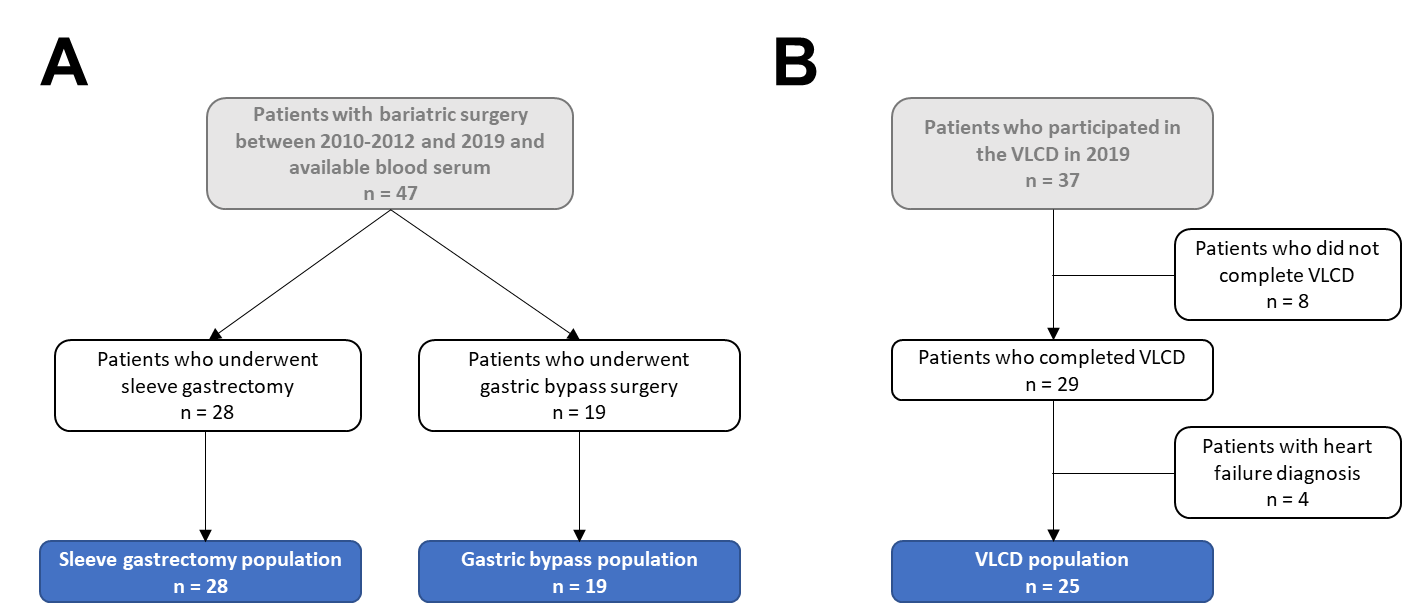


(A) Patients who underwent bariatric surgery between 2010-2012 and in 2019, and who had available blood serum left for measurement of NT-proBNP, were included in the analysis (n=47). Of these patients, n=28 underwent sleeve gastrectomy and n=19 underwent gastric bypass surgery. None of these patients had a diagnosis of heart failure. (B) In 2019, n=37 patients participated in the VLCD weight loss program at the interdisciplinary center of obesity medicine of the University Hospital Schleswig-Holstein (Kiel, Germany). Out of these patients, n=8 did not complete the program and n=4 had a diagnosis of heart failure. Thus, n=25 patients were included in the VLCD group of this present study.

## Supplemental Figure 2 – Study design of the intervention groups


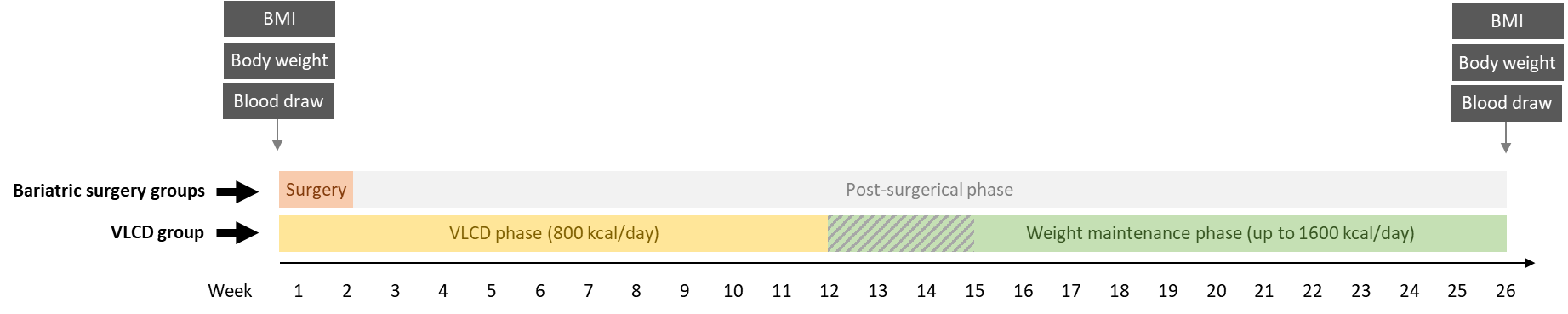


All patients underwent a fasting blood draw and a body weight and height measurement at baseline, e.g. before surgery and before start of the VLCD. Patients in the surgery groups underwent laparoscopic sleeve gastrectomy or Roux-en-Y gastric bypass surgery and were examined by a surgeon 3 months post-surgery. After 6 months, blood draws and body weight and height measurements were repeated. Patients in the VLCD group were given an approximately 800 kcal/day formula-based and micronutrient-balanced diet for 3 months with a subsequent weight maintenance phase of further 3 months, where they received up to 1600 kcal/day. The shaded area denotes the transition time between VLCD and weight maintenance, where individuals have been slowly adapted to a regular diet. After 6 months, blood draws and body weight and height measurements were repeated. This figure was created with Microsoft PowerPoint 2019 (16.0.13127.21062) 64-Bit Edition (Link: <https://www.microsoft.com/de-de/microsoft-365/powerpoint>)

## Supplemental Figure 3 – Study flowchart of the cross-sectional group


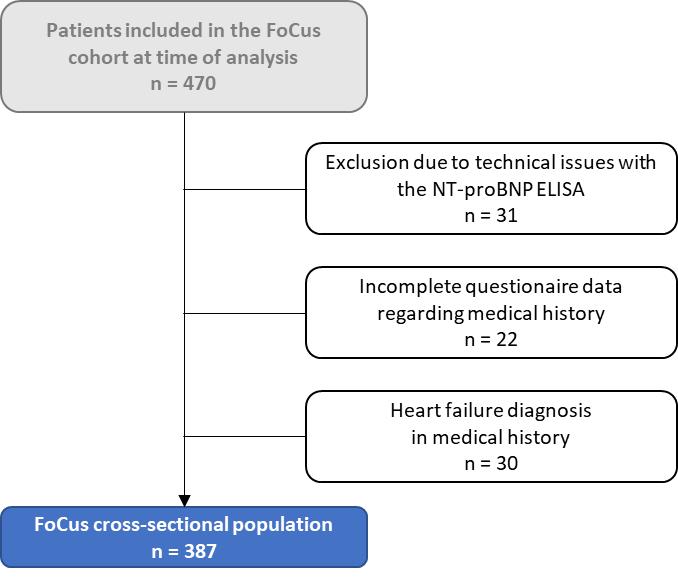


At time of analysis, n = 470 individuals have been included in the FoCus cohort. Of those individuals, n=31 were excluded to due technical issues with the NT-proBNP ELISA kit, n=22 were excluded to due incomplete questionnaire data regarding their medical history, and n=39 were excluded due to a preexisting heart failure diagnosis. In the final cross-sectional analysis, n=387 individuals were included.

## Supplemental Figure 4 – Association between weight loss and the reduction in fasting blood glucose after a 26-week weight loss program.

The Pearson’s correlation coefficient was used to quantify associations between continuous variables. VLCD, very low-calorie diet.

## Supplemental Figure 5 – Comparison of changes in NT-proBNP concentration after sleeve gastrectomy, gastric bypass surgery, and a 26-week weight loss program including values below the ELISA detection limit.

Bars denote median NT-proBNP concentration and error bars denote 95% CI of the median. Y axes are formatted in log^10^ to account for skewed distribution of NT-proBNP values. This analysis includes NT-proBNP values below the detection limit of the ELISA (sleeve gastrectomy: n=28, gastric bypass n=19, VLCD: n=25). Statistical significance of changes in NT-proBNP concentration from pre- to post-intervention was determined by Wilcoxon signed rank test. CI; confidence interval; NT-proBNP, N-terminal pro brain natriuretic peptide; VLCD, very low-calorie diet.
